# Exposure to War-Related Stress and Suicidal Ideation: The Moderating Role of Interpersonal and Political Trust

**DOI:** 10.3390/bs16060887

**Published:** 2026-06-01

**Authors:** Eliane Sommerfeld

**Affiliations:** Department of Psychology, Ariel University, Ariel 4070000, Israel; sommerfelde@ariel.ac.il

**Keywords:** suicide, exposure to war-related stress, interpersonal trust, political trust

## Abstract

Exposure to war-related stress is associated with increased risk for suicidal ideation, yet the role of different domains of trust under conditions of ongoing collective threat remains insufficiently understood. This study examined the association between exposure to war-related stress and suicidal ideation and tested whether interpersonal, political, and institutional trust moderated this association. Participants were 367 adults living under ongoing war-related conditions in Israel who completed self-report measures of exposure to war-related stress, trust, and suicidal ideation. Greater exposure to war-related stress was associated with higher suicidal ideation. Interpersonal trust was associated with lower suicidal ideation, whereas political and institutional trust were not. Moderation analyses showed domain-specific effects: interpersonal trust buffered the association between exposure and suicidal ideation, such that the association weakened and became non-significant at higher levels of interpersonal trust. In contrast, political trust strengthened this association, with higher exposure more strongly associated with suicidal ideation among individuals reporting higher political trust. Institutional trust showed no significant main or moderating effects. Findings suggest that trust domains play distinct roles in suicidal ideation during war. Interpersonal trust may be protective, whereas political trust may be associated with increased vulnerability under conditions of exposure to war-related stress.

## 1. Introduction

Armed conflict constitutes a major risk context for psychological distress and suicidal ideation. Exposure to war-related stress may include direct threat to life, bereavement, displacement, injury, military mobilization, disruption of daily routines, and indirect exposure through media and social networks. Such conditions can undermine basic assumptions regarding safety, predictability, and the reliability of social and political institutions, thereby increasing vulnerability to depressive and suicidal states.

The present study examines these processes in the context of the war in Israel that began following the attacks on 7 October 2023. The attacks and subsequent war exposed Israeli civilians to varying degrees of direct and indirect war-related stress, including violence, loss, displacement, missile attacks, reserve military service, and prolonged disruption of daily life. Emerging evidence indicates that this period was accompanied by marked increases in depression, anxiety, and post-traumatic stress symptoms among Israeli civilians (Groweiss et al., 2024; Levi-Belz et al., 2024; Yamin et al., 2025). Within this context, the present study focuses on suicidal ideation and asks whether interpersonal, institutional, and political trust moderate the association between exposure to war-related stress and suicidal ideation.

Exposure to war-related violence has been repeatedly associated with heightened psychological distress in both civilian and military populations (Lim et al., 2022). Research on trauma and mental health has traditionally focused on post-traumatic stress disorder (PTSD) as a primary consequence of war exposure (Hoppen et al., 2021; Kienzler, 2008). However, accumulating evidence suggests that other outcomes warrant equal attention in conflict-affected populations, particularly depression and suicidality (Cogo et al., 2022; Jankovic et al., 2013; Snir et al., 2017). Depression and suicidal ideation may arise from war-related trauma through sustained fear, a diminished sense of communal safety, a loss of control, and erosion in trust in institutions (Brewin et al., 2025). Notably, these responses are not confined to individuals with PTSD. Depression and suicidal ideation can develop along trajectories that are partially independent of PTSD, reflecting distinct psychological mechanisms (Wang et al., 2023). Furthermore, both PTSD and depression independently predict suicidality (Brewin et al., 2025; Ramsawh et al., 2014), and certain trauma dimensions, such as combat-related killing, are associated with increased suicide risk even in the absence of pronounced PTSD symptoms (Khan et al., 2023).

The association between war and suicide is complex and may vary according to the type, duration, and stage of conflict. Historical and sociological accounts suggest that suicide rates may sometimes decline during wars, possibly because collective threat temporarily increases social integration and shared purpose (Durkheim, 1897/1951; Lester, 1994; Aida, 2020). However, prolonged exposure to war-related stress may increase vulnerability, probably because of the post-war onset of mental disorders (Karam et al., 2012). This complexity highlights the need to examine moderators that may strengthen or weaken the association between exposure to war-related stress and suicidal ideation.

Although war-related trauma is consistently associated with increased psychological distress, exposure alone does not fully explain variations in mental health outcomes. Individual differences in psychological responses remain substantial, and research increasingly highlights the role of psychological and social resources in adaptation to traumatic events (Bonanno et al., 2023). Social–cognitive resources, including beliefs and expectations that shape how people interpret and engage with their social environment, are thought to influence both vulnerability and resilience during periods of intense stress (Bonanno et al., 2024).

Among social resources, trust in other people and in societal systems has emerged as an important determinant of resilience. In broad terms, trust reflects expectations that others will act fairly and cooperatively and that public institutions will operate competently and in line with shared norms and values (Nannestad, 2008; Zhao et al., 2024). Prior work commonly distinguishes between several forms of trust. Generalized interpersonal trust refers to beliefs about the reliability and goodwill of people in general and shapes expectations of social support and mutual aid (Nannestad, 2008). Political trust reflects confidence in political actors and governing processes, including the expectation that decision-makers act with integrity and in the public interest (Zmerli & Hooghe, 2013). Institutional trust concerns evaluations of the legitimacy and effectiveness of specific public institutions (e.g., governmental bodies, security forces, courts) that play a central role during periods of collective threat (Sønderskov & Dinesen, 2016).

Trust may moderate the association between exposure to war-related stress and suicidal ideation because it shapes how individuals interpret threat, evaluate available support, and locate themselves within interpersonal and collective systems. Interpersonal trust may buffer the psychological impact of war-related stress by increasing expectations that others are reliable, supportive, and available in times of danger. Individuals with higher interpersonal trust may therefore be more likely to seek help, mobilize social support, and maintain a sense of belonging, processes that are central to theoretical accounts of suicide risk emphasizing thwarted belongingness and social disconnection (Joiner et al., 2005; Van Orden et al., 2010). Institutional trust may also buffer distress by reinforcing confidence that public systems remain capable of providing protection, continuity, and access to essential resources during crises.

Political trust may play a different and more complex moderating role. Trust in political actors and governing processes may provide a sense of order and collective confidence during war, but it may also heighten expectations that leaders and public systems will provide protection and stability (Rose et al., 2011; Devine et al., 2021). When such expectations are frustrated during prolonged crisis, disappointment, betrayal, or loss of meaning may intensify the psychological impact of exposure to war-related stress (Smith & Freyd, 2017).

Although trust is often conceptualized as a relatively stable propensity shaped by personality, early socialization, and cultural context, it also has a state-like dimension that can shift in response to lived experiences and sociopolitical events (Dinesen & Bekkers, 2017). This dynamic aspect is particularly relevant during war and collective violence, when exposure to threat, loss, and social disruption can wear down trust in both people and institutions (Conzo & Salustri, 2019; Fiedler, 2023). Trust may erode after mass violence due to perceptions of betrayal or abandonment by authorities, evaluations of delayed or ineffective institutional responses, and direct observation of moral transgressions by others (Hall & Werner, 2022; Fiedler, 2023). Reduced interpersonal trust may weaken perceived social support and community solidarity (Kawachi & Berkman, 2001), while reduced political and institutional trust are often associated with heightened cynicism, alienation, and feelings of helplessness (Rothstein & Stolle, 2008). Conversely, higher trust across these domains has been associated with better mental health outcomes following disasters, partly because trust facilitates cooperation, information sharing, and access to material and social resources (Sibley et al., 2020). In the context of the ongoing war, where governmental responses have been uneven and publicly contested, trust may therefore be a particularly salient factor in explaining individual differences in suicidal ideation.

Despite growing evidence that war-related stress is associated with psychological distress and suicide-related outcomes, less is known about the psychosocial conditions that may shape the strength of this association. In particular, trust may be especially relevant during periods of collective threat because it reflects individuals’ expectations regarding the reliability of other people, public institutions, and political systems. However, relatively little research has examined whether different forms of trust moderate the association between exposure to war-related stress and suicidal ideation. Addressing this gap is important because it may help identify not only individuals who are more vulnerable to suicidal ideation during war but also the relational and social resources that may buffer or amplify this risk.

### The Present Study

This cross-sectional study examined whether trust moderates the association between exposure to war-related stress and suicidal ideation among Israeli civilians approximately six months after the attacks on 7 October 2023, during an ongoing conflict. By focusing on trust as a social–cognitive factor that shapes how individuals interpret threat, evaluate available support, and relate to interpersonal and political systems, the present study contributes to the growing literature on psychosocial factors associated with suicidal ideation in contexts of collective trauma. Based on prior research, we hypothesized that (a) higher exposure to war-related stress would be associated with higher levels of suicidal ideation; (b) higher interpersonal, political, and institutional trust would be associated with lower levels of suicidal ideation; and (c) trust would moderate the association between exposure to war-related stress and suicidal ideation.

In the present study, exposure to war-related stress was operationalized as the cumulative number of self-reported war-related stress experiences endorsed by participants. Suicidal ideation was operationalized as the frequency of self-reported suicidal thoughts during the previous two weeks. Interpersonal trust was operationalized as general trust in other people’s fairness, honesty, and willingness to help. Political trust was operationalized as trust in the values presumed to guide political and public decision-making, including honesty, openness, and fairness. Institutional trust was operationalized as confidence in major public institutions in Israeli society.

## 2. Materials and Methods

### 2.1. Participants and Procedures

Participants were recruited using a non-probability convenience sampling approach through social media platforms and a professional online sampling service. Social media recruitment invited Israeli young adults to participate in an anonymous online study examining psychological responses during the ongoing war. In addition, the professional sampling service distributed the survey to eligible participants from its participant pool according to predefined eligibility criteria. Inclusion criteria were age 18–40 years, residence in Israel at the time of data collection, and ability to complete the questionnaire in Hebrew. No additional exclusion criteria were applied at the recruitment stage. Responses were excluded from the final analytic sample if the questionnaire was incomplete or if responses did not meet basic data-quality criteria, such as completion times that were implausibly short for attentive questionnaire completion. An a priori power analysis was conducted using G*Power v. 3.1.9.7 for linear multiple regression, a fixed model, and R^2^ deviation from zero. Assuming a medium effect size (f^2^ = 0.15), α = 0.05, statistical power of 0.80, and 7 predictors in the final regression model, the required sample size was 103 participants. The final sample exceeded this requirement and consisted of 367 young adults (49.9% women), residing in Israel, aged 18–36 (M = 25.23, SD = 2.89). Most participants were single (69.2%); smaller proportions were married (24.5%), divorced or widowed (1.9%), or reported another relationship status (4.4%). Years of education ranged from 10 to 22 (M = 13.7, SD = 2.1). Most participants (68.9%) were either fully or partially employed. Approximately half (50.4%) described their economic status as average, 31.3% as below or far below average, and 18.3% as above or far above average. Most participants (93.2%) identified as Jewish, while 6.8% identified as Muslim, Christian, Druze, or other. In terms of religiosity, 41.1% identified as secular, 25.3% as traditional, 19.6% as religious, and 12.3% as very religious.

The study was approved by the Institutional Review Board of Ariel University. Informed consent was obtained from all participants. Data were collected online using Qualtrics (Qualtrics, Provo, UT, USA). After reading the informed consent form, participants completed self-report questionnaires assessing demographic characteristics, exposure to war-related stress, suicidal ideation, and interpersonal, political, and institutional trust. Participation was anonymous and voluntary, and participants could discontinue participation at any stage. The questionnaire included information about psychological support services in case participants experienced distress or wished to seek professional help.

### 2.2. Instruments

#### 2.2.1. Suicidal Ideation

Suicidal ideation was assessed using the Paykel Suicide Scale (Paykel et al., 1974), a brief self-report measure of suicidal feelings and thoughts. The scale includes five items: the first four assess suicidal feelings and suicidal ideation of increasing severity, whereas the fifth assesses suicide attempts. The measure has been employed in multiple epidemiological studies (e.g., Skoog et al., 1996; Rosta & Aasland, 2013). It has shown adequate internal consistency and test–retest reliability indices (Fonseca-Pedrero & Pérez de Albéniz, 2020). In addition, extensive research supports its construct, convergent, and divergent validity, with studies demonstrating a robust unidimensional factor structure, significant positive correlations with depression and anxiety, and negative correlations with protective factors such as self-esteem and perceived social support (Fonseca-Pedrero et al., 2018; Séris-Martínez et al., 2026; Sterud et al., 2008). Because the present study focused on suicidal ideation rather than suicidal behavior, only the first four items were included in the ideation score. This approach is consistent with previous studies that have treated the first four Paykel items as an index of suicidal ideation or suicidal feelings, while considering the fifth item separately as assessing suicide attempts (e.g., Berg et al., 2003; Fässberg et al., 2020; Göbbels-Koch, 2024). Participants rated how frequently they had experienced each thought over the past two weeks on a 6-point scale ranging from 1 (never) to 6 (all the time). Internal consistency for the four-item ideation score was high (Cronbach’s α = 0.88). A total score was computed by summing the item responses, with higher scores indicating greater suicidal ideation. The score was used as a continuous index of suicidal ideation; no clinical cutoff or normative classification was applied.

#### 2.2.2. Exposure to War-Related Stressors

Exposure to war-related stress was assessed using the checklist of war-related stressors (Ho et al., 2023), which drew on the war-exposure checklist used by Karatzias et al. (2023) in their study of war exposure among people living in Ukraine during the Russian war. The checklist assesses stressful civilian experiences during war, including direct exposure to danger, displacement, economic damage, injury or death of close others, damage to property and infrastructure, and difficulties accessing basic resources. The measure was selected for the present study because it assesses broad civilian experiences of war-related stress rather than events unique to one national context. For the present study, the Hebrew items were reviewed and adapted to the Israeli context of the 7 October war and its aftermath. The adapted version included 28 dichotomous items assessing exposure to a range of war-related stressors, including disruption of daily life, forced separation, displacement, war-related threat, missile or bombing exposure, participation in or exposure to combat, injury or loss, captivity or abduction, and death. Items were coded as 1 = yes and 0 = no and were summed to create a total exposure score ranging from 0 to 28, with higher scores indicating greater exposure to war-related stress. Because the measure reflects cumulative exposure to distinct war-related stressors rather than a unidimensional latent construct, internal consistency was not calculated. However, the use of a checklist-based cumulative exposure score is consistent with the purpose of the measure, namely, to capture the breadth of exposure to war-related stressors.

#### 2.2.3. Trust in Interpersonal, Political, and Institutional Domains

Trust was assessed using 13 items selected and adapted from previous studies and measurement recommendations in the trust literature (Joxhe, 2022; Sønderskov & Dinesen, 2016). The items were not developed as a new psychometric scale but were used as brief indices of three theoretically distinct forms of trust relevant to the present study: interpersonal trust, political trust, and institutional trust. This distinction is consistent with prior literature differentiating trust in other people from trust in political actors and public institutions. The items were grouped into three subscales reflecting distinct dimensions of trust. Items 1–3 assessed interpersonal trust, referring to general perceptions of people’s fairness, honesty, and willingness to help (Sønderskov & Dinesen, 2016). Items 4–6 evaluated political trust, defined as trust in the core values presumed to guide public institutions, specifically, honesty, openness, and fairness (Joxhe, 2022). Items 7–13 measured institutional trust by asking participants to indicate their level of confidence in seven major public institutions in Israeli society: the Knesset (Israeli parliament), the municipality, the government, the courts, the police, the Israel Defense Forces (IDF), and the media (Sønderskov & Dinesen, 2016). All items were rated on a scale from 0 to 10, where 0 indicated no trust at all and 10 indicated the highest possible level of trust. In the present sample, internal consistency was acceptable to high: interpersonal trust (α = 0.80), political trust (α = 0.91), and institutional trust (α = 0.74). For each subscale, a mean score was calculated, with higher scores reflecting greater trust in that domain.

#### 2.2.4. Demographic Information

Participants provided information regarding their age, gender, marital status, number of children, years of education, place of birth, and location on 7 October 2023 (name of city or town). They were also asked to report their religious affiliation, nationality, employment status, and subjective socioeconomic status. Subjective socioeconomic status was assessed using a single self-report item asking participants to rate their economic status relative to the average in Israel. Responses were provided on a five-point ordinal scale ranging from 1 = far below average to 5 = far above average, with higher scores indicating higher perceived socioeconomic status.

### 2.3. Data Analysis

Descriptive statistics, correlations, and regression analyses were performed using SPSS v. 29. Because subjective socioeconomic status was measured on a five-point ordinal scale, associations involving socioeconomic status were examined using Spearman’s rho. Pearson correlations were used for associations among the continuous study variables. Although gender was not a primary variable of interest, *t*-tests for independent samples were conducted exploratorily to examine gender differences in study variables.

To examine whether exposure to war-related stress and trust dimensions were associated with suicidal ideation and whether trust moderated the association between exposure and suicidal ideation, a hierarchical multiple regression analysis was conducted with suicidal ideation as the dependent variable. Demographic variables were considered as potential covariates but were not included in the primary regression model because they did not meet empirical criteria for confounding the main associations. Specifically, they were not consistently associated with both the predictors and suicidal ideation. The analysis followed a three-step procedure. In Step 1, exposure to war-related stress was entered. In Step 2, the three trust variables were entered: interpersonal trust, political trust, and institutional trust. In Step 3, the interaction terms between exposure and each of the trust variables were entered. Sensitivity analyses were conducted with age, gender, and subjective socioeconomic status entered as covariates; the substantive pattern of findings remained unchanged. Significant interactions were further examined using the regression-based PROCESS supplement to SPSS (Model 1) (Hayes, 2022), with bootstrapping based on 5000 bootstrap samples. Predictors and moderators were centered prior to all regression analyses. Effect sizes were reported using Pearson’s r for bivariate correlations among continuous variables, Spearman’s rho for associations involving socioeconomic status, Cohen’s d for gender comparisons, R^2^ and ΔR^2^ for regression models, and standardized regression coefficients for individual predictors. For moderation analyses, ΔR^2^ was used to evaluate the incremental variance explained by the interaction terms.

## 3. Results

### 3.1. Descriptive Statistics and Preliminary Analyses

Before testing the main study hypotheses, descriptive statistics and preliminary gender comparisons were examined. Descriptive statistics for the full sample and by gender are presented in Table 1. *t*-tests for independent samples revealed that men reported significantly higher exposure to war-related stress [t = 3.42, df = 363, *p* < 0.001], as well as higher levels of political trust [t = 4.04, df = 363, *p* < 0.001] as compared to women. No significant gender differences were found for suicidal ideation, interpersonal trust, or institutional trust.

### 3.2. Correlational Analyses

To address the first and second hypotheses, bivariate correlations were examined between exposure to war-related stress, trust dimensions, and suicidal ideation. Bivariate correlations are presented in Table 2. Greater exposure to war-related stress was associated with increased suicidal ideation. However, exposure showed no associations with participants’ trust levels. Suicidal ideation was negatively correlated with interpersonal trust, while political and institutional trust were not associated with suicidal ideation. Among demographic variables, higher economic status was associated with lower levels of suicidal ideation. Age was negatively associated with institutional trust, suggesting that younger participants reported greater institutional trust.

### 3.3. Regression Analyses

To address the third hypothesis, which proposed that trust would moderate the association between exposure to war-related stress and suicidal ideation, three interaction terms were entered into the final step of the hierarchical regression model: Exposure × Interpersonal Trust, Exposure × Political Trust, and Exposure × Institutional Trust. The results are presented in Table 3. In Step 1, exposure to war-related stress accounted for a small but statistically significant proportion of the variance in suicidal ideation. Greater exposure to war-related stress was associated with higher levels of suicidal ideation. In Step 2, the three trust variables were added, and they significantly improved the model. Interpersonal trust emerged as a significant negative predictor of suicidal ideation, whereas political trust and institutional trust were not significant predictors. In Step 3, the interaction terms significantly improved the model. Two interactions were significant: interpersonal trust buffered the association between exposure and suicidal ideation, whereas political trust amplified this association. The Exposure × Institutional Trust interaction was not significant. Overall, the final model accounted for 10.8% of the variance in suicidal ideation.

**Figure 1:**
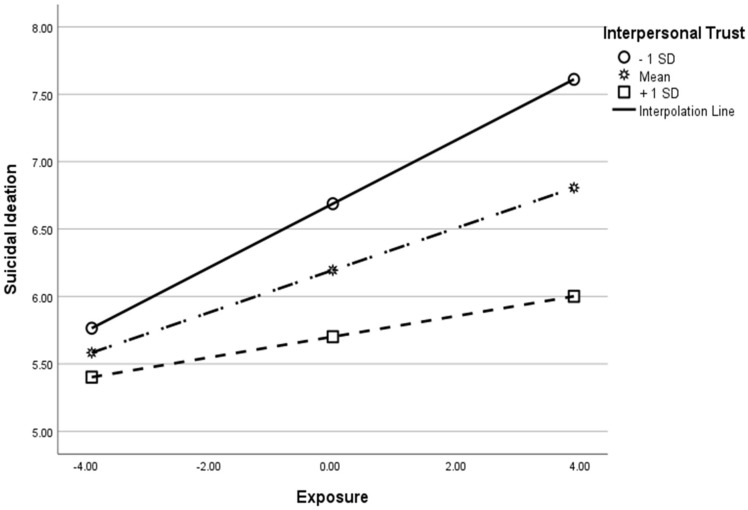
Interpersonal trust as moderator in the relationship between exposure and suicidal ideation.

**Figure 2:**
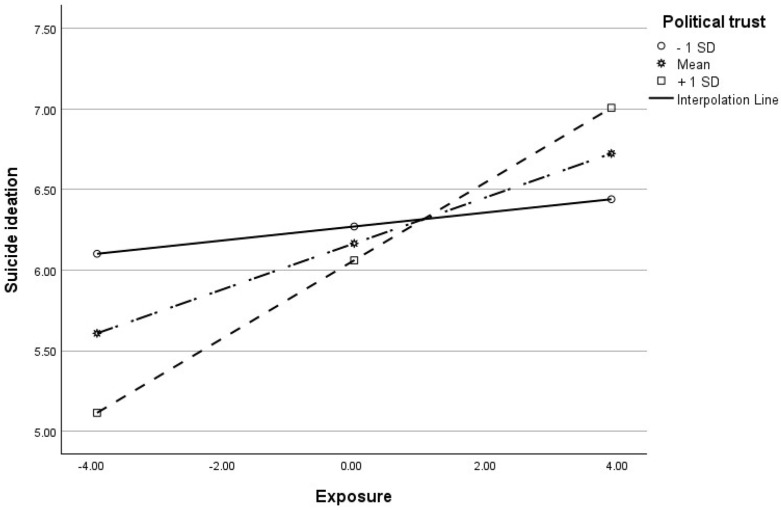
Political trust as moderator in the relationship between exposure and suicidal ideation.

Because the Exposure × Interpersonal Trust and Exposure × Political Trust interactions were significant, simple slopes analyses were conducted for these two interactions using PROCESS Model 1, with predictors centered prior to the analyses. The Exposure × Institutional Trust interaction was not significant and was therefore not further probed. The results are presented in Table 4 and Figure 1 and Figure 2. A significant interaction emerged between exposure to war-related stress and interpersonal trust in predicting suicidal ideation (b = −0.041, SE = 0.019, *p* = 0.036). Simple slopes indicated that exposure was positively associated with suicidal ideation at low levels of interpersonal trust (−1 SD) and at mean levels, but not at high levels of interpersonal trust (+1 SD). These results indicate that the association between exposure and suicidal ideation weakens as interpersonal trust increases.

For political trust, a significant interaction emerged between exposure to war-related stress and political trust in predicting suicidal ideation (b = 0.042, SE = 0.018, *p* = 0.021). Simple-slopes analysis indicated that exposure was not associated with suicidal ideation at low levels of political trust (−1 SD) but was significantly associated at mean levels and showed an even stronger association at high levels of political trust (+1 SD), indicating that political trust amplifies the association between exposure and suicidal ideation, such that individuals with higher political trust exhibit a stronger positive relationship between exposure and suicidal ideation.

## 4. Discussion

This study examined how interpersonal, political, and institutional trust relate to suicidal ideation among Israeli civilians during the war that began on 7 October 2023. As hypothesized, greater exposure to war-related stress was associated with higher levels of suicidal ideation. Interpersonal trust emerged as a protective factor, which was associated with lower levels of suicidal ideation, whereas political and institutional trust were not significantly related to suicidal ideation. Notably, interpersonal and political trust moderated the relationship between exposure to war-related stress and suicidal ideation, but in opposite directions. High interpersonal trust attenuated this association, whereas high political trust amplified it.

We found that greater exposure to war-related stress was associated with higher levels of suicidal ideation. Comparable patterns have been reported in studies from other conflict settings (Cogo et al., 2022; Charlson et al., 2019; Panter-Brick et al., 2018). Taken together, these findings indicate that exposure to violence and ongoing threat is associated with increased mental distress and suicidal ideation. However, the modest association observed in the present study should also be interpreted in light of evidence that the relationship between war and suicide is not uniform. Prior research suggests that suicide rates may sometimes decline during acute war, possibly because collective threat temporarily strengthens social integration and shared purpose, whereas war exposure may also increase vulnerability to suicidality through war-related mental disorders and persistent psychological distress (Durkheim, 1897/1951; Lester, 1994; Aida, 2020; Karam et al., 2012). Prolonged exposure to war-related stress may increase psychological vulnerability through chronic threat, uncertainty, loss, and erosion of perceived safety (Hobfoll et al., 2007; Miller & Rasmussen, 2010). In the present study, the significant interaction effects suggest that this association depends partly on psychosocial context: interpersonal trust weakened the association between exposure and suicidal ideation, whereas political trust strengthened it. Thus, rather than suggesting a simple or uniform association between war-related stress and suicidal ideation, the present findings support the need to examine psychosocial moderators, such as interpersonal and political trust, that may shape this association.

In the present study, interpersonal trust was the only trust dimension consistently associated with lower levels of suicidal ideation. This aligns with prior studies linking higher interpersonal trust to better psychological well-being and lower suicide risk under conditions of prolonged uncertainty and threat (Costanza et al., 2021). Interpersonal trust also moderated the association between exposure and suicidal ideation. At higher levels of trust, exposure was not significantly related to suicidal ideation, whereas at lower levels of trust, greater exposure was associated with higher ideation. This pattern suggests that interpersonal trust may shape how individuals manage social relationships under conditions of prolonged threat. Individuals with higher interpersonal trust may be more likely to perceive others as supportive and reliable, which could facilitate help-seeking, mobilization of social support, and the maintenance of hope during extreme adversity (Thoresen et al., 2018). In turn, trust may reduce feelings of isolation, strengthen perceptions of shared fate, and enhance a sense of belonging (Costanza et al., 2021; Yu et al., 2019). Taken together, these findings are in line with theoretical models that emphasize social connectedness and perceived belonging as key buffers against distress following exposure to war-related stress (Joiner et al., 2005; Maercker & Horn, 2013).

In contrast, political trust strengthened the association between exposure and suicidal ideation. Among individuals reporting high political trust, greater exposure was associated with higher levels of suicidal ideation, whereas no such association was observed among those with lower levels of trust. One possible interpretation is that strong political trust may heighten sensitivity to disappointment or disillusionment when political actors or systems fail to meet expectations during crises (Frateur et al., 2025; Zmerli & Hooghe, 2013). Unmet expectations regarding governmental competence and fairness, such as perceived delays in rescue efforts or inadequate crisis management, may give rise to feelings of betrayal, loss of meaning, and heightened distress (Aassve et al., 2024; Ungar, 2013). This interpretation is consistent with institutional betrayal theory, which posits that reliance on authorities that subsequently fail to provide protection can amplify psychological harm (Christl et al., 2024; Smith & Freyd, 2017). By contrast, when expectations of political institutions are lower, institutional shortcomings may be experienced as less personally salient, potentially reducing their psychological impact (Proszowska et al., 2023). At the same time, low political trust is not without cost, as it has been linked to reduced civic engagement, lower adherence to public safety measures, and decreased willingness to seek institutional support (Devine et al., 2021). Over time, such disengagement may weaken collective responses and contribute to broader social fragmentation. Future research could examine when political trust is associated with better mental health outcomes and when it may increase vulnerability following exposure to war-related stress.

In the present study, institutional trust was not significantly associated with suicidal ideation, nor did it moderate the effects of exposure. This finding suggests that in acute phases of armed conflict, generalized confidence in institutional functioning may play a less central role in immediate psychological outcomes than more proximal, relational forms of trust, such as trust in other people. It remains possible that institutional trust may assume greater importance in later phases of recovery or reconstruction. This possibility warrants further empirical investigation.

Overall, the results point to the value of distinguishing between different forms of trust when examining psychological responses to exposure to war-related stress. Interpersonal trust appears to serve a protective role, in line with theoretical models and research studies emphasizing social buffering and resilience. Political trust, by contrast, appears to interact with trauma exposure in more complex ways and, under certain sociopolitical conditions, may be linked to increased vulnerability. Understanding these nuanced relationships is essential for the development of psychosocial interventions. Programs aiming to strengthen interpersonal trust and social connectedness may be particularly effective in mitigating suicide risk in populations affected by war and terrorism. From a policy perspective, these findings suggest that public trust depends not only on cultivating positive attitudes but also on visible accountability, transparency, and responsiveness during crises. Clinically, attention to both interpersonal and political trust may help identify individuals who differ in their vulnerability following exposure to collective threat.

### 4.1. Implications

The findings have several clinical, psychosocial, and policy implications. The protective role of interpersonal trust suggests that suicide-prevention efforts during prolonged exposure to war-related stress should not focus only on individual symptoms but also on strengthening relational and community resources. Existing interventions, such as gatekeeper training, peer-support programs, psychological first aid, crisis-helpline services, and community-resilience programs, could be adapted to address interpersonal trust more explicitly. For example, such programs may include components that encourage help-seeking, reduce barriers to disclosing suicidal thoughts, strengthen perceived social availability, and train community members, educators, and clinicians to respond reliably and nonjudgmentally to distress.

Second, the finding that political trust amplified the association between exposure to war-related stress and suicidal ideation suggests that collective and political meanings may shape individual distress during war. Clinicians working with individuals exposed to war-related stress may need to attend not only to fear and trauma symptoms but also to experiences of disappointment, betrayal, moral injury, anger toward authorities, or loss of confidence in political systems. These themes may be especially salient when individuals have strong expectations that political actors or public systems would provide protection, stability, or moral clarity.

Finally, at the policy level, the findings suggest that suicide prevention during prolonged exposure to war-related stress should be integrated into broader public-health and community-resilience strategies. Policymakers should ensure that mental-health services are accessible, visible, and proactively offered to populations exposed to ongoing threat. Concrete steps may include transparent public communication, visible accountability, proactive outreach to highly exposed groups, coordination among mental-health services, municipalities, educational institutions, workplaces, and community organizations, and clear referral pathways to psychological and social support. Such policy efforts may be especially important during prolonged conflict, when distress is shaped not only by direct exposure to danger but also by uncertainty, disruption of daily life, and perceived failures of protection or responsiveness.

### 4.2. Limitations

Because the study was cross-sectional, causal inferences cannot be drawn, and it remains possible that suicidal ideation influences levels of trust rather than the reverse. Longitudinal research is needed to clarify temporal relationships. In addition, all measures were based on self-report and may be influenced by social desirability. Moreover, no formal psychometric validation has yet been reported for the war-related exposure checklist used in the present study. Although the measure was adapted from previous research on war-related stressors (Ho et al., 2023; Karatzias et al., 2023; Smidt et al., 2024), future studies should further examine its psychometric properties, including its content validity, test-retest reliability, and associations with related measures of trauma exposure, psychological distress, and suicidality across different war-exposed populations and sociopolitical contexts. Another limitation concerns the measurement of trust. Although the trust items were selected and adapted from previous studies and measurement recommendations, the present study did not conduct a full validation of the adapted trust indices. Internal consistency was examined separately for interpersonal, political, and institutional trust; however, future research should further examine their content validity, factor structure, test–retest reliability, convergent validity, and discriminant validity in Israeli samples exposed to war-related stress. Finally, the study was conducted during an ongoing conflict, and the findings may not generalize to post-conflict or peacetime contexts. Future research should explore the dynamic interplay between trust and mental health over time, including potential reciprocal influences. Qualitative studies could clarify how individuals interpret trust disruptions or trust maintenance during war and whether these processes vary by cultural or political context. Additionally, research could test whether intervention programs designed to strengthen interpersonal trust also reduce suicidality following exposure to war-related stress.

## 5. Conclusions

The present findings indicate that different forms of trust are differentially associated with psychological responses to exposure to war-related stress. Distinct patterns observed for interpersonal and political trust underscore the importance of differentiating between trust domains when examining suicide risk in times of war. From a social psychiatric perspective, these results highlight trust as a salient social–cognitive factor shaping individual vulnerability under conditions of sustained collective threat. Future research should further examine how trust-related processes interact with contextual and relational factors to inform suicide prevention efforts in populations exposed to war.

## Figures and Tables

**Table 1:** Descriptive statistics and gender differences for study variables.

		Total (N = 367) ^a^	Men (N = 182)	Women (N = 183)		
Variable	Range	M (SD)	M (SD)	M (SD)	d	t (363)
Suicidal ideation	4–24	6.19 (3.38)	5.99 (3.38)	6.37 (3.37)	−0.11	−1.08
Exposure	0–22	5.84 (3.90)	6.53 (4.74)	5.15 (2.70)	0.36	3.42 ***
Interpersonal trust	0–10	4.87 (2.06)	4.98 (2.10)	4.77 (2.03)	0.10	0.98
Political trust	0–10	2.68 (2.28)	3.15 (2.32)	2.21 (2.14)	0.42	4.04 ***
Institutional trust	0–9.14	3.93 (1.71)	4.04 (1.90)	3.83 (1.51)	0.12	1.15

Note: ^a^ Two participants did not report their gender. d = Cohen’s d; negative values indicate higher scores for women. *** *p* < 0.001.

**Table 2:** Correlations between the study variables (N = 367).

Variable	1	2	3	4	5	6	7
1. Suicide Ideation	---						
2. Exposure	0.17 ***	---					
3. Interpersonal Trust	−0.15 **	0.00	---				
4. Political Trust	−0.03	0.04	0.33 ***	---			
5. Institutional Trust	−0.07	−0.04	0.39 ***	0.65 ***	---		
6. Gender	0.06	−0.18 ***	−0.05	−0.21 ***	−0.06	---	
7. Age	0.05	0.00	−0.06	−0.05	−0.24 ***	−0.17 ***	---
8. Economic Status	−0.13 *	−0.06	0.09	0.01	0.07	−0.14 *	−0.06

Note: * *p* < 0.05, ** *p* < 0.01, *** *p* < 0.001. The associations between economic status and the other variables were examined by Spearman’s Rho.

**Table 3:** Hierarchical regression analysis predicting suicidal ideation.

Predictor	*B*	*SE B*	*β*	*t*	*p*
Exposure	0.15	0.05	0.17	3.34	*p* < 0.001
*R*^2^ = 0.030, Δ*R*^2^ = 0.030, *F* (1, 365) = 11.17, *p* < 0.001					
Exposure	0.15	0.05	0.17	3.33	*p* < 0.001
Interpersonal Trust	−0.25	0.09	−0.15	−2.75	*p* < 0.01
Political Trust	0.04	0.10	0.03	0.41	*p* > 0.05
Institutional Trust	−0.04	0.14	−0.02	−0.30	*p* > 0.05
*R*^2^ = 0.054, Δ*R*^2^ = 0.024, *F* (3, 362) = 3.05, *p* < 0.05					
Exposure	0.13	0.04	0.15	2.94	*p* < 0.01
Interpersonal Trust	−0.28	0.09	−0.17	−3.11	*p* < 0.01
Political Trust	0.06	0.10	0.04	0.57	*p* > 0.05
Institutional Trust	−0.03	0.13	0.02	−0.25	*p* > 0.05
Exposure × Interpersonal	−0.08	0.02	−0.21	−3.78	*p* < 0.001
Exposure × Political	0.06	0.02	0.17	2.69	*p* < 0.01
Exposure × Institutional	0.04	0.03	0.09	1.36	*p* > 0.05
*R*^2^ = 0.108, Δ*R*^2^ = 0.054, *F* (3, 359) = 7.29, *p* < 0.001					

**Table 4:** Conditional effects of exposure on suicidal ideation at low (−1 SD), mean, and high (+1 SD) levels of interpersonal and political trust.

Moderator Level	*B*	*SE*	*t*	*p*	95%CI
Interpersonal trust					
−1 SD	0.23	0.06	3.93	*p* < 0.001	[0.12, 0.35]
Mean	0.15	0.04	3.41	*p* < 0.001	[0.06, 0.24]
+1 SD	0.07	0.06	1.11	*p* > 0.05	[−0.05, 0.18]
Political trust					
−1 SD	0.04	0.07	0.61	*p* > 0.05	[−0.09, 0.17]
Mean	0.14	0.04	3.03	*p* < 0.01	[0.05, 0.22]
+1 SD	0.23	0.06	4.09	*p* < 0.001	[0.12, 0.34]

Note. SD = standard deviation; CI = confidence interval.

## Data Availability

The datasets generated during the current study are not publicly available due to ethical restrictions related to participant confidentiality but are available from the corresponding author upon reasonable request.
